# A Typical Case of Multisystem Inflammatory Syndrome in a 10-yearold Girl with COVID-19: A Case Report from Ethiopia

**DOI:** 10.4314/ejhs.v32i4.26

**Published:** 2022-07

**Authors:** Mikyas Demissie, Solomie Jebessa Deribessa, Tigist Bacha

**Affiliations:** 1 Pediatrician at Ethio-Tebib General Hospital, Addis Ababa, Ethiopia. mikyasdemissie@gmail.com; 2 Pediatrics Infectious Disease Specialist at St.Paul's Hospital Millennium Medical college, Addis Ababa, Ethiopia solomejebessa@gmail.com, sdsj40@gmail.com; solomie.jebessa@sphmmc.edu.et; 3 Pediatrics Emergency and critical care specialist at St.Paul's Hospital Millennium Medical college, Addis Ababa, Ethiopia. tigistbacha@yahoo.com

**Keywords:** Severe Acute Respiratory Syndrome Corona Virus-2, COVID-19, Multisystem inflammatory syndrome

## Abstract

**Background:**

Severe acute respiratory syndrome-Corona Virus -2 (SARS-CoV2) has infected more than 500 million and has claimed the lives of more than 6.1 million people worldwide.

**Case:**

We are presenting a 10-year-old girl who fulfilled the criteria of Multisystem inflammatory disease associated with COVID-19(MIS-C). She had fever of > 3 days, muco-cutaneous lesions, hypotension/shock, myocardial dysfunction, acute gastrointestinal symptoms, elevated markers of inflammation, coagulopathy without other microbial causes and positive COVID RT-PCR test.

**Conclusions:**

When pediatric patients present with the above symptoms and signs we should have a high index of suspicion of MIS-C for timely action and better outcome.

## Introduction

The Severe Acute Respiratory Syndrome CoronaVirus-2 (SARS-CoV2) has infected more than 500 million and has claimed the lives of over 6.1 million people worldwide, and in Africa over 8.6 million people were infected and over 171 thousand have died. In Ethiopia the number of cases has raised to more than 470 thousand and 7509 people have died (1).

Children and adolescents below 20 years of age account for up to 18% of COVID-19 cases and 0.4% of deaths. The majority (58%) of cases in children were between 10 and 19 years of age(2).

Starting early 2020, cluster of children and adolescents have been indentified exhibiting multisystem inflammatory condition with some features similar to Kawasaki disease (KD) and toxic shock syndrome (TSS) Later on, clinical and cytokine profile studies identified that though there were some overlapping features between KD (with TSS) and MIS-C, there were some distinctions in their biochemical response (3).

Acute COVID-19 in children present initially with respiratory symptoms, where as multisystem inflammatory syndrome (MIS-C) occurs in few patients and is a progressive illness and commonly present with fever, abdominal pain and /or rash advancing to multisystem involvement with higher morbidity and mortality.

The exact reason why some children exhibit MIS-C and others do not is unknown, but it is presumed to be an exaggerated immune response to COVID-19 (4).

In May 2020, considering the cluster of cases from Europe and North America, the World Health Organization defined MIS-C in children and adolescents 0–19 years of age as: a fever lasting ≥ 3 days along with two of the following (5):

- Rash or bilateral non-purulent conjunctivitis or muco-cutaneous inflammations (oral, hands and feet);
- Hypotension/shock;
- Features of myocardial dysfunction, pericarditis, valvulitis or coronary abnormalities, elevated Troponin/ N-terminal-pro-brain natriuretic peptide;
- Evidence of coagulopathy (by Partial thromboplastin time (PTT), prothrombin time (PT) and elevated d-Dimers); and
- Acute gastrointestinal problems (diarrhea, vomiting or abdominal pain).

The above, along with elevated markers of inflammation such as erythrocyte sedimentation rate (ESR), C-reactive protein (CRP) or procalcitonin, as well as 1) evidence of COVID-19 infection with a real time Reverse Transcription Polymerase Chain Reaction [RT-PCR) antigen test or serology positive] or 2) likely contact with COVID-19 patients but with no other obvious microbial causes of inflammation particularly staphylococcal or streptococcal shock syndrome, is diagnostic of MIS-C(5). Since then, several case reports, case series and surveillance reports have been released from different countries; Nigeria, South Africa and Ethiopia. We are reporting the case of a 10 year old girl who presented in February 2021 with symptoms of MIS-C and had been a diagnostic and management challenge in our setting.

## Case Presentation

A 10-year-old girl presented with headache, fever, and easy fatigability of 5 days, associated with vomiting, and a generalized rash lasting 2 days. She did not have a previous history of drug allergy or chronic illness. She had received all childhood vaccines.

**Figure 1 a and 1b:**
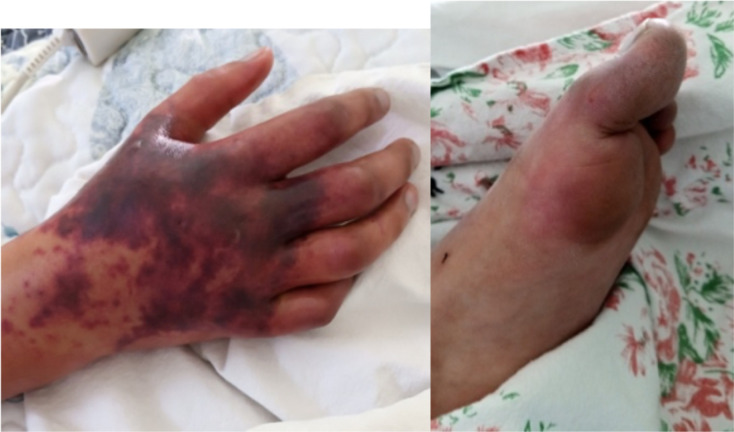
Dark discoloration of fingers with dark red patch at the back of the right hand and left foot and toes of the 10 year old girl with MIS-C, on day 7 of admission; February, 2021.

On physical examination: her BMI was 13.7 <5th percentile (CDC BMI chart for her age). She had a fever of 39 degrees, her blood pressure ranging between 60/40 to 90/50 (with I.V fluid resuscitation), her tonsils were swollen red with exudates and her right anterior cervical lymph nodes were enlarged. Two days after admission she developed decreased air entry in her lungs bilaterally, abdominal tenderness on epigastric area and multiple erythematous, macular skin lesions over the trunk and extremities. On seventh day these advanced to dark discoulorations of right hand, fingers and all toes (Figure 1a,1b). Initially she was conscious without neurologic deficit but progressively deteriorated and lapsed in to coma.

**Laboratory investigation** (Tables 1) revealed persistently elevated white blood cells with neutrophilia and raised ESR. Her blood culture had no bacterial growth. She had hyponateremia, raised serum creatinine, sterile pyuria, and albuminuria. Her liver enzymatic and synthetic functions were raised twice the normal values on the third day, and became 10 times on day five. Between the third and fifth day, serum albumin and total protein dropped further. Her HIV, Hepatitis B, Hepatitis C and anti nuclear antibody (ANA) tests were all negative. Her D-dimers, Lactate-dehydrogenas (LDH) and CRP were all markedly elevated.

**Table 1:** Blood workups of a 10 year old girl with Multi-system Inflammatory Syndrome, Addis Ababa, Ethiopia, February, 2021

Lab tests [N= normal values for age]		1^st^ day	2^nd^	3^rd^	4^th^	5^th^	6^th^	7^th^	8^th^	9^th^	10^th^
WBC: [N=(4–10.5)*1000] Neutrophil 54- 62%		15000, *Neu=91%		26,400 Neu= 84 %			24100 Neu=85.7%	18300 Neu=81%	14,200 Neu=86%	14.200 Neu=87.2%	
Blood culture				No growth							
Hemoglobin gm/dl[N=12–15]				12.89			12.1	11.8	10.2	10.7	
Platelet[N=(150–400) *1000]				184,000			100,000	59,000	34,000	52,000	
†ESR [N=up to 30 mm/hr]		70									
‡COVID-19 Tests (§RT-PCR)			Negative								Positive
Serum electrolyte	Na mmol/L [135–145]	128		137							
	K+ mmol/L[3.3–4.6]			4.39							
	Cl mmol/L[98–106]			110							
Liver Function:	AST U/L[ N=5–30]			65		262		135	474		
	ALT U/L[N=5–45]			41		269		238	590		
	ALP								287		
	PT			21.9				43.3	27.7	21.8	
	PTT			31.6				41	37.9	30.5	
	INR			2.01				4.15	2.65	2	
Renal function	Creatinin [Nl=0.31–0.88]	0.89	1.47	1.39		1.12					
	Urea [N=5–18]	84				110					
Serum Albuming/dl [N=3.5–5.6]				2.9		2.3		2			
Total Protein mg/dl [N=6.4–8.1]				4.3		4.2		3.9			
Other blood tests:		HIV antibody, Hepatitis B surface antigen (HBSAg), Hepatitis C anti body (HC-Ab) and qualitative anti nuclear antibody (ANA) tests were all negative;									
D-dimers [≤ 500 ng/ml] Fibrinogen equivalent unit (FEU)		2857.5 ng/ml									
Lactate dehydrogenas (LDH) [N=120–330 U/L]		1183U/L									
C-reactive protein (CRP) [N=0.06–0.79 mg/dl]		20.6mg/dl									

*Neu=Neutrophils; †ESR=Erythrocyte Sedimentation Rate; ‡COVID-19=Corona disease-2019, §RT-PCR= Real Time Reverse Transcription Polymerase Chain Reaction

**Figure 2:**
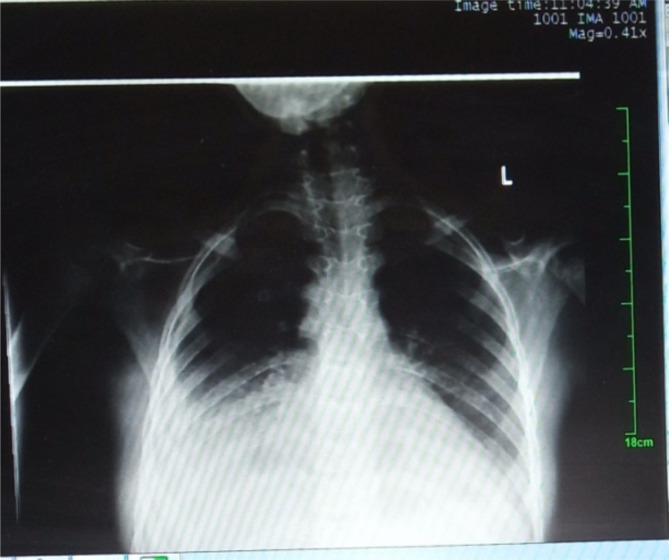
chest x-ray of the 10 year old girl with MIS-C : bilateral pleural effusion with collapsed basal lung segments (more on the right), February, 2021.

Her abdominal ultrasound (Table 2) showed mesenteric lymphadenitis, bilateral renal parenchymal echogenecity, hypoechoic hepatomegaly. Her chest X -ray revealed bilateral pleural effusion and cardiomegaly (Figure 2). Her pleural fluid was transudate and peritoneal fluid showed high WBC count with neutrophil predominance; however both fluids were negative for gram stain or acid-fast stains. Her Echocardiography was indicative of dilated cardiomyopathy. Doppler ultra-sound revealed multiple right upper and lower digital arterial tree occlusive microthrombi.

**Table 2:** Imaging results of a 10-year-old girl with Multi-system Inflammatory Syndrome. Addis Ababa, Ethiopia, February, 2021

Imaging	Result
Ultrasound of the abdomen	Mesentric lymphadynopathy, bilateral renal paranchymal echogenecity; Liver span of 14.4 cm, slightly hypoechoic with slight prominence of hepatic veins, small volume of ascites
Chest x-ray	1^st^ Chest X-ray: Bilateral pleural effusion with collapsed basal lung segments 2^nd^ Chest X-ray: Cardiomegaly (done on 8^th^ day)
Echocardiography	All cardiac chambers were dilated, left ventricular ejection fraction of 22% and fiber shortening fraction of 7 %, right ventricular function was grossly reduced. Assessment: dilated cardiomyopathy following acute myocarditis with severe biventricular dysfunction.
Doppler ultra sound	Multiple right digital arterial tree, left distal posterior tibialis and bilateral dorsalis pedis hypoechoic intraluminal filling defect likely occlusive septic thrombo emboli. No sonographic abnormality was seen in the upper and lower extremities venous ultrasound.

**Treatment and course**

Initially she was treated for acute pyelonephritis, sepsis and mild hyponatremia. On the third day she developed cough, respiratory distress and hypotension; with an echocardiography result of biventricular dysfunctions; uncontrolled sepsis with multi-organ involvement was considered. Antibiotics were revised to Vancomycin and Meropenum and she was managed with diuretics and inotropes.

On day-four she had signs of impending respiratory failure with persistent hypotension, she was intubated and put on synchronized intermittent mandatory ventilation (SIMV). She was resuscitated with I.V fluids and then treated with Adrenaline and then Dopamine. On the fifth and sixth days, she was in decompensated shock despite continuous efforts and on day- seven, she developed disseminated intravascular coagulation (DIC) with gangrenous digits. She was transfused with fresh frozen plasma and platelet concentrate, Enoxaparin was started. A second COVID RT-PCR sample was taken (COVID-19 antibody test was not available). She was started on Dexamethason, Asprin and intravenous immunoglobulin (IVIG).

On the ninth day, she became unconscious with pin pointed reactive pupils, stroke due to DIC was considered but brain imaging was not possible due to her critical condition. She succumbed on the next day, with possible cause of death attributed to multi-organ failure following MIS-C. The second RT-PCR test for COVID 19 was reported as positive after her death.

## Discussion

Our patient fulfilled all the criteria of MIS-C. She had a fever ≥ 3 days and muco-cutaneous inflammation. She was repeatedly treated for hypotension/shock, had myocardial dysfunction, acute gastrointestinal symptoms with elevated markers of inflammation and coagulopathy without other microbial causes (with negative blood and urine cultures), and positive COVID RT-PCR test (5).

Pediatric patients with MIS-C commonly present with fever, abdominal pain and /or rash, like our patient (2,3).

In our patient the initial negative RT-PCR test for COVID-19 in lieu of lack of COVID-19 antibody test created unnecessary delay in the start of specific management for MIS-C. Postmortem examination was not sought for this patient. The diagnostic and management challenges we faced informed us that we should have a high clinical index of suspicion for MIS-C in this COVID era for timely action and better outcome.
